# Variations in Methodological Approaches to Measuring Health Inequalities and Inequities: A Scoping Review of Acute Stroke Pathways

**DOI:** 10.3390/healthcare13121410

**Published:** 2025-06-12

**Authors:** Stephen McCarthy, Peter McMeekin, Michael Allen, Martin James, Anna Laws, Andrew McCarthy, Graham McClelland, Lisa Moseley, Laura Park, Daniel Phillips, Christopher Price, Jason Scott, Lisa Shaw, Phil White, David Wilson, Gary A. Ford

**Affiliations:** 1 Faculty of Health and Life Sciences, Northumbria University, Newcastle upon Tyne NE1 8ST, UK; peter.mcmeekin@northumbria.ac.uk (P.M.); andrew2.mccarthy@northumbria.ac.uk (A.M.); graham.mcclelland@northumbria.ac.uk (G.M.); laura.j.park@northumbria.ac.uk (L.P.); jason.scott@northumbria.ac.uk (J.S.); 2 Medical School, University of Exeter, Exeter EX1 2LU, UK; m.allen@exeter.ac.uk (M.A.); martinjames@nhs.net (M.J.); a.laws2@exeter.ac.uk (A.L.); 3 NIHR South West Peninsula Applied Research Collaboration (ARC), Plymouth EX1 2LU, UK; 4 East of England Ambulance Service NHS Foundation Trust, Cambridgeshire SG8 6EN, UK; daniel.phillips@eastamb.nhs.uk; 5 Stroke Research Group, Population Health Sciences Institute, Newcastle University, Newcastle upon Tyne NE3 3LZ, UK; c.i.m.price@newcastle.ac.uk (C.P.); lisa.shaw@newcastle.ac.uk (L.S.); phil.white@newcastle.ac.uk (P.W.); 6 Stroke Service User Voice Group, Newcastle upon Tyne, UK; dave.5.wilson2118@gmail.com; 7 Oxford University Hospitals NHS Foundation Trust, Oxford OX3 9DU, UK; gary.ford@ouh.nhs.uk; 8 Division of Medical Sciences, University of Oxford, Oxford OX1 2JD, UK

**Keywords:** stroke, health inequality, prehospital

## Abstract

Background: There are a lot of advances that may affect the way treatment is delivered prehospital, including mobile stroke units and point-of-care diagnostics. These have the potential to affect populations differently and therefore affect the distribution of health outcomes. Objectives: We aimed to address the following research questions: (1) Which geographic and socioeconomic inequalities have been included when evaluating access to acute stroke treatment (including reperfusion therapies)? (2) How have the identified measures been considered/assessed/calculated? (3) We also report any methodological approaches that have been proposed that might further improve the way in which acute stroke care interventions are analysed, specified relating to inequalities. Methods: PubMed and Scopus electronic databases were searched for studies that included participants who underwent acute stroke treatment and included quantitative measures of geographic and/or socioeconomic inequalities or inequities in accessing/receiving treatment. Results: Overall, sixty-six studies were included in the review. Fifty-nine included at least one measure of geographic inequalities or inequities while thirty-six included at least one measure of socioeconomic inequalities or inequities. Twenty-eight of these studies included both a geographic and socioeconomic measure of inequalities or inequities. There were no commonalities in the methods of defining, categorising and measuring the inequalities or inequities. No study provided their definition of inequality or inequity or stated any normative judgements they had made. Conclusions: It is vital that the evaluation of programmes like acute stroke care consider impacts on inequality and inequity. Researchers and policy makers should work together to determine relevant measures of inequality/inequity and the most appropriate methods of measuring and categorising them. In addition, researchers should make it clear within their work how they are defining inequality and inequity and what (if any) normative judgements have been made.

## 1. Background

Since the mid-1990s, the optimal treatment for acute ischaemic stroke has been emergency admission to hospital with possible restoration of cerebral blood supply by reperfusion therapy where appropriate. Initially, intravenous tissue plasminogen activator (tPA) was the only therapeutic option, but more recently, endovascular interventions such as mechanical thrombectomy (MT) have become viable therapeutic options at the most highly resourced facilities [1]. For eligible patients, the primary goal of such therapies is to restore blood flow quickly and effectively to the affected area and minimise irreversible tissue damage. This is dependent on rapid recognition of stroke symptoms and having efficient ways to assess and transport a patient to a hospital able to provide specialist stroke care [1]. In the United Kingdom and elsewhere, care providers and researchers have spent considerable time and resources on improving outcomes via earlier identification of eligible patients and by reducing the time it takes to initiate reperfusion treatment for ischaemic stroke.

Treating stroke patients as soon as possible makes the link between geography and treatment outcomes inevitable, as therapy for patients residing further from treatment centres will begin later than for patients nearer to treatment. Health systems moving towards centralised stroke care may lead to better overall outcomes [2] but could also have an impact on geographical inequalities, although interventions such as mobile stroke units (modified ambulances containing brain imaging equipment and stroke practitioners) may address this. Intersectionality, as a metaphor, describes compounded inequalities; this applies to the geographic inequalities of time taken to access therapy, which is frequently accompanied by socioeconomic inequalities observed in more remote populations [3]. In addition, socioeconomic disparities exist in stroke hospitalisation risk, case fatality and the quality of healthcare [4]. Despite initiatives to reduce inequalities, there are still methodological issues that need addressing in the evaluation of structural and therapeutic changes to acute stroke care in terms of their effects on inequalities. These issues are associated with the way in which geographic and socioeconomic inequalities are often measured as a distribution across a continuous scale, and how different distributions are compared.

Health inequality is the generic term used to designate differences, variations, and disparities in the health status of individuals and groups, while health inequity refers to those inequalities in health that are deemed to be unfair or stemming from some form of injustice [5]. While health inequalities may exist due to voluntarily assumed risks, pure chance, or life stage differences, these are not normally considered unjust and therefore are not a health inequity. For example, random genetic mutations or age differences may lead to health inequalities but are not considered to stem from an unfairness or injustice (although societal attitudes to these resulting in health inequalities may be considered an unfairness or injustice) [5]. However, health inequalities due to class or race are generally considered to be unfair and an injustice, and therefore are not only a health inequality but a health inequity. Health inequalities, including those that result from how healthcare is delivered, are best explained by the structural theory of inequalities [6], which states that socioeconomic circumstances of groups cause differences in health outcomes, and thus systematic disparities are embedded in the organisational and institutional aspects of healthcare systems. Faced with limited resources, addressing health inequities can involve the redistribution of healthcare resources at the costs of reducing overall efficiency. Policymakers and providers may have to consider where resources should be targeted to treat the greatest number or the greatest need.

In addition, within health inequity, there exist the concepts of horizontal inequity and vertical inequity. Cookson et al. define horizontal inequities as unequal treatment for equal groups, while vertical inequities are defined as equal treatment for unequal groups [7]. Vertical equities are, therefore, appropriate unequal treatment for unequal groups. From these concepts, it follows that, in terms of access to acute stroke treatments, a health inequality that is not due to unfairness or injustice may be an example of vertical health equity. That is, appropriate unequal access for unequal groups could be measured as a health inequality.

The aim of this review was to identify the methods in which geographical and socioeconomic inequalities when accessing acute stroke treatment (including reperfusion therapies) are accounted for in the wider literature, for the purpose of informing evaluations of new interventions or ways of working, such as mobile stroke units. Specifically, we aimed to address the following research questions: (1) Which geographic and socioeconomic inequalities have been included when evaluating access to acute stroke treatment (including reperfusion therapies)? (2) How have the identified measures been considered/assessed/calculated? (3) Might any methodological approaches that have been proposed further improve the way in which acute stroke care interventions are analysed, specified relating to inequalities? This paper presents a discussion of how methodological developments in the discipline of economic evaluation could be applied by researchers to the evaluation of acute stroke care pathways and outcomes in any health system so that inequalities are given appropriate consideration.

## 2. Methods

### 2.1. Study Design

Scoping reviews are a valuable tool to explore and clarify complex ideas and can be useful when planning research [8]. We conducted a scoping review summarising the available evidence on how socioeconomic and geographic inequalities/inequities in accessing acute stroke treatment are currently considered [8]. The Preferred Reporting Items for Systematic reviews and Meta-Analyses extension for Scoping Reviews (PRISMA-ScR) was used to structure the review, with the PRISMA-ScR checklist available in the Supplementary Material S3 [9].

### 2.2. Eligibility Criteria

Studies were included in this review if they included participants who underwent acute stroke treatment and included quantitative measures of geographic and/or socioeconomic inequalities or inequities in accessing/receiving treatment. Geographical measures of inequality/inequity included rurality, distance or time to hospital and geographic region, with socioeconomic measures of inequality/inequity including income levels, medical insurance status and education level. Observational studies and randomised trials were included. Measures of sex, race, age and other inequalities/inequities were not included as they are outside the scope of this review, despite being intersectional with geographic and/or socioeconomic inequalities/inequities. However, studies that included these factors alongside geographic or socioeconomic factors were included.

### 2.3. Information Sources

PubMed and Scopus electronic databases were searched. The search strategy (Supplementary Material S1) was adapted as appropriate for each database to allow for variations in controlled vocabulary terms and syntax [10]. The searches were conducted on 30 April 2024. Studies included in systematic reviews that satisfied the inclusion criteria of this review were also screened for eligibility. Hand searching of the references of included studies was also conducted.

### 2.4. Study Selection

All titles and abstracts were independently screened by two reviewers. The full text of selected studies was independently assessed by two investigators and classified as relevant, not relevant or unclear according to the inclusion criteria. Disagreements at either stage were resolved through discussion and, where necessary, a third reviewer arbitrated.

### 2.5. Data Collection Process

A standardised bespoke data collection form was developed to collect data from included studies. Data were extracted by one reviewer and all data were checked by a second reviewer for accuracy. Disagreements were resolved by discussion or arbitration by a third reviewer. Data were extracted for study aim(s), study design, area of interest, and data source, along with information regarding the type of inequality or inequity measured, method of measurement, and reasoning behind measurement.

### 2.6. Data Analysis

Data collected from each eligible study were collated by inequality or inequity type (geographic or socioeconomic) and were grouped into relevant categories. Within each category, the method of measuring and/or categorising each inequality or inequity was recorded, allowing common themes or methods to be identified and synthesised narratively. In addition, the source of the data for each study was collected to understand the impact of similar data sources on variable choice and measurement.

## 3. Results

**Figure 1:**
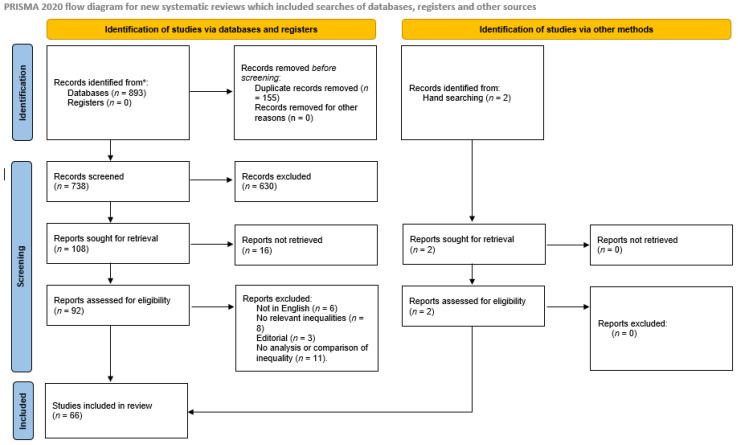
Synthesis of results.

The searches identified 738 unique records with an additional 2 records identified from hand searching (Figure 1). Of these, 674 were not eligible for inclusion in this review. Publications identified were published from 1996 onwards (to 2024). Ineligibility was primarily due to the studies not including geographic or socioeconomic inequalities or inequities. Overall, 66 studies were included in the review. Studies came from 20 countries, with papers from the USA being the most common (*n* = 23), followed by Spain (*n* = 4), France (*n* = 4), Australia (*n* = 4) and South Korea (*n* = 4). Two studies took a global perspective.

**Figure 2:**
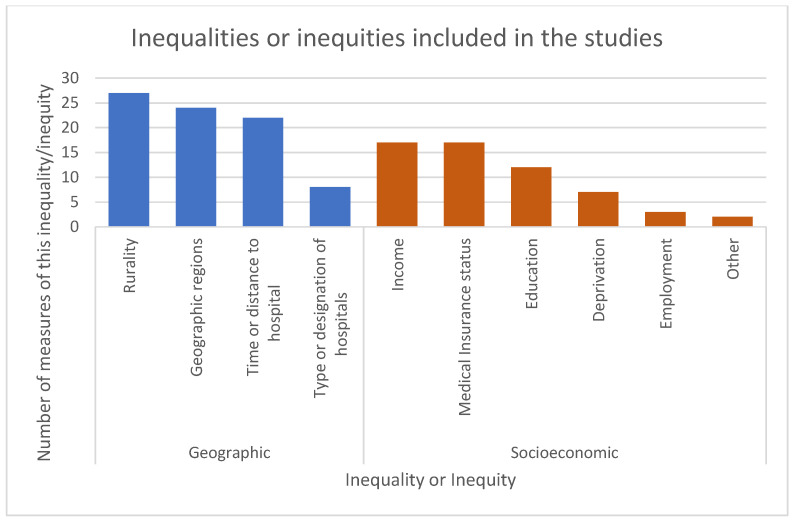
Inequalities or inequities included in the studies [11,12,13,14,15,16,17,18,19,20,21,22,24,26,27,28,29,30,32,33,34,35,36,37,38,39,40,43,44,45,46,47,48,49,50,51,52,53,55,56,57,58,59,60,61,62,63,64,65,66,67,69,70,71,72,73,74,75,76].

Of the 66 included studies [11,12,13,14,15,16,17,18,19,20,21,22,23,24,25,26,27,28,29,30,31,32,33,34,35,36,37,38,39,40,41,42,43,44,45,46,47,48,49,50,51,52,53,54,55,56,57,58,59,60,61,62,63,64,65,66,67,68,69,70,71,72,73,74,75,76], 59 included at least one measure of geographic inequalities or inequities [11,12,13,14,15,16,17,18,19,20,21,22,24,26,27,28,29,30,32,33,34,35,36,37,38,39,40,43,44,45,46,47,48,49,50,51,52,53,55,56,57,58,59,60,61,62,63,64,65,66,67,69,70,71,72,73,74,75,76], while 36 included at least one measure of socioeconomic inequalities or inequities [12,13,17,18,20,21,22,23,24,25,26,28,31,35,41,42,44,45,46,48,49,51,52,53,54,55,58,62,63,64,68,69,70,71,72,74] (Supplementary Table S1). Twenty-nine of these studies included both a geographic and socioeconomic measure of inequalities or inequities [12,13,17,18,20,21,22,24,26,28,35,44,45,46,48,49,51,52,53,55,58,62,63,64,69,70,71,72,74]. A total of one hundred and thirty-nine measures of geographic or socioeconomic inequalities or inequities were included within the studies (Table 1 and Figure 2).

### 3.1. Geographical Inequalities

A total of eighty-one measures of geographic inequalities or inequities were included within fifty-nine studies. Forty studies included only a single measure of geographic inequalities or inequities [11,13,14,17,18,19,20,21,22,26,27,28,29,30,33,36,37,38,39,40,44,47,48,49,50,52,53,56,58,59,60,61,64,65,69,71,72,73,76], sixteen studies included two separate measures [12,15,16,24,34,45,46,55,57,62,66,67,70,74,75], and three studies included three measures [32,63]. Of the 81 measures of geographic inequalities or inequities included, 69 were measured on a categorical scale, while 12 were continuous.

The most common measure of geographic inequality or inequity was rurality, which was described in twenty-seven variables. Twenty-four of these were categorical, with fourteen of these reporting a dichotomous measurement, urban compared to rural (or similar wording). No study that used a dichotomous measurement provided their definition of urban and/or rural. Four studies that used a non-dichotomous measurement of rurality gave their category boundaries, but no study explained how or why these boundaries had been chosen. Three measurements were continuous, two reporting people per square mile/km and one study reporting the percentage of the population in each region that was rural. The next largest category of measures was “geographic regions”, such as regions of the USA [12,32,45,46,51,55,66,67,74,75,76] or North India compared to South India [61], with twenty-four variables, all being categorical. The third most common measure was the distance or time to hospital, reported in twenty-two variables. Thirteen of these measures were categorical and nine were continuous, with the continuous variables being measured as either miles/km or minutes. Finally, there were eight categorical inequalities or inequities that measured the type or designation of hospitals.

### 3.2. Socioeconomic Inequalities

Fifty-eight measures of socioeconomic inequalities or inequities were reported within thirty-six studies. Nineteen studies included a single measure of socioeconomic inequality or inequity [13,17,18,20,21,22,23,26,31,41,49,58,62,63,64,68,69,70], thirteen studies included two separate measures [12,24,35,44,45,46,48,51,52,53,54,55,74], three studies included three measures [25,71], and one study included four measures [72]. Of the fifty-one measures of socioeconomic inequalities or inequities included, forty-four were measured on a categorical scale, while seven were continuous.

There were seventeen measures of the medical insurance status of the patient, all reported categorically [12,18,24,26,31,35,44,45,46,51,52,53,54,55,68,70,74]. There were also seventeen measures of the income of the patient, fourteen of which had this measure banded into categories [12,24,25,35,45,46,51,52,53,54,55,64,71,74], while three studies included this as a continuous variable [23,63,72]. Education was also a common measure of socioeconomic inequality, with ten measures being categorical and two measures being continuous [13,17,22,25,28,41,42,48,49,69,71]. For the two continuous variables, one study measured the years of school [22], and another measured the percentage of the population with a bachelor’s degree [72]. Seven studies included a measure of deprivation (six categorical, one continuous) [20,21,44,48,58,62,72] and three studies included employment (two categorical, one continuous (percentage of the population in employment)) [25,71,72]. One study also included two measures of socioeconomic inequality or inequity that was unique to that study (if the patient had a telephone (categorical) and if the patient received a certain benefit (categorical) [42].

In the seventeen studies that included income as a socioeconomic inequality or inequity, ten were US studies that used the National Inpatient Sample, a large publicly available all-payer inpatient care database, reporting income in quartiles [77]. Four studies reported income as a categorical variable, using different approaches (such as one study using above average, below average and poverty level), with none explaining the rationale for the approach used. Amongst the two studies that included income as a continuous variable, one used Gross Domestic Product (Purchasing Power Parity) per capita [72] and the other used annual wage level (in CNY) [63]. Seventeen studies reported medical insurance status: fifteen were based in the USA and therefore used categories of US medical insurance status, and not all are applicable outside the USA. Ten studies included education as a categorical variable, using nine different methods of categorisation.

## 4. Discussion

It is well recognised that socioeconomic and geographic inequalities and inequities contribute to the risk and burden of stroke; however, the effects of inequalities and inequities in access to acute stroke treatment are less well understood [4]. To understand these effects clearly, researchers require clear definitions of how the potential inequalities and inequities are measured and categorised, an explanation of how inequalities and inequities are being defined, and an acknowledgement of any normative judgements that are being made (where a normative judgement refers to a value judgement, exploring what should or ought to be).

In this scoping review of sixty-six studies, we found that measurement of geographic and socioeconomic inequalities in access to acute stroke treatment is heterogeneous, with little commonality between studies. Studies that included similar measures of inequality generally used their own methods of defining, categorising and, critically, measuring the inequality. Even in those studies that included rurality as a binary variable, no study included their definition of rurality, leading to a lack of clarity as to whether these definitions are consistent across studies and creating challenges for generalisation of evidence. Even studies that focus on the same country can use different methods of categorisation. The exception to this is eleven studies which all included income and medical insurance status, with similar categories for both [12,35,45,46,51,52,53,54,55,66,74]. However, all eleven of these studies were in the same country (USA) and all used the same data source. These differences in variable choice and measurement choice could be due to a variety of reasons such as limits to data availability (as per the eleven USA studies noted above), researcher choice or decision, or differences in the study area or population. However, it should be noted that one of the key findings of this review is that reasons behind variable choice were rarely explicitly stated.

Given the lack of clear international guidelines or conventions in including or categorising geographic and socioeconomic inequalities to evaluate access to acute stroke treatment, future research should align with the relevant decision maker’s own expectations. This may differ between systems where, for example, publicly funded systems might have a greater expectation to actively reduce inequalities and inequities than systems that rely more on private funding. For example, within the UK, the English NHS has the Core20PLUS5 agenda [78]. This agenda aims to reduce healthcare inequalities at both the national and system level by targeting the most deprived 20% of the population, identified by the Index of Multiple Deprivation, a composite measure of disadvantage that includes seven domains [79]. In addition, the agenda identifies PLUS population groups, who should be identified at a local level. Researchers should examine if any of these groups or if any other disadvantaged groups (specific to the health system being examined) exist within their study population that require focused attention. For example, in the USA, Social Determinants of Health are frequently used [80]. In addition, several measurements of geographic or socioeconomic inequality or inequity (such as medical insurance status or measures of deprivation) can be country-specific and have little relevance or meaning to an international audience. Therefore, it should be incumbent on authors to provide sufficient background for and justification of methodological decisions regarding the selection of variables to allow readers to understand the context of their work, and to allow the meaning of the evidence created to be more directly applicable to real-world settings.

Although our search criteria included both inequalities and inequities, the majority of studies included described their results in terms of inequalities (or in similar terms such as disparities or differences). To distinguish an inequality as an inequity requires making a normative (value) judgement on whether the underlying cause is a form of unfairness or injustice [5]. Only ten studies [13,20,27,29,36,46,55,67,73,74] in this review made a normative judgement and characterised the differences they observed between the groups in their population as inequities, therefore implicitly stating that these differences are due to unfairness or injustice between the groups. The remaining 56 studies reported only the existence and scale of the inequality and did not make judgements as to the fairness of the underlying cause, leaving this to policy makers and the reader. In addition, when incorporating the concepts of horizontal and vertical inequity, a second normative judgement must be made in order to report inequities: that this is an example of horizontal inequity and not an example of vertical equity (that is, a judgement that the difference between the groups is an inequity because the groups are equal and should receive equal treatment). It is not an example that the groups are unequal and are receiving (appropriately) unequal treatment.

However, no studies within this review have explicitly stated what (if any) normative judgements they have made. This leads to a lack of clarity for the reader, who is left to detect these judgements by themselves, and a lack of clarity over the study’s results. Without knowing the definitions of inequality and/or judgements used, it is unclear as to whether any differences with the study populations are due to a health inequality (which may or may not be due to an unfairness or injustice), a health inequity (which is due to an unfairness or injustice) or a health inequality that is also a vertical health equity (that is, appropriate unequal access or outcomes within the population that is due to unequal needs). For example, eleven studies used the same data source, the American National Inpatient Sample [77]. All eleven of these studies reported outcome differences between populations of different income, medical insurance status, rurality, and geographic region. However, eight of the studies reported these as inequalities and made no judgements on if the cause of these inequalities was due to unfairness or injustice [12,35,45,51,52,53,54,66]. The three remaining studies reported inequities, indicating that they made two normative judgements: (1) these differences are due to unfairness or injustice and (2) this is not an example of vertical equity (i.e., the study populations are equal and should receive equal treatment (but are receiving unequal treatment)) [46,55,74]. While it may seem relatively straightforward to make these normative judgements, the ethical and societal implications may be far-reaching. The concept of ‘personal health responsibility’ has led to claims that patients with ‘self-inflicted’ conditions have less of a right to treatment at the public’s expense than patients whose conditions arose from ‘uncontrollable’ causes [81]. Depending on the nature of the healthcare system, this could mean that inequities (which are due to unfairness) are considered to make more of a right to public funds than some inequalities. These normative judgements of a difference in inequity could be seen as the researcher (advertently or inadvertently) making this claim.

In addition, within the studies, it was unclear as to whether the researchers themselves were clear about their definitions of inequality and inequity and what (unstated) normative judgements they had made. Twenty-nine of the papers used the word disparity to describe any differences within their population. While, under our definition, a disparity is a health inequality, it may also be taken to mean a health inequity (particularly in an American context) [7]. Furthermore, thirteen papers used a combination of terms, mixing inequalities, inequities, and disparities within their manuscripts [21,23,24,25,27,28,29,36,51,53,63,67,69]. This introduces further confusion about any normative judgements the authors may have made and if they consider the differences within their populations to be the result of unfairness or injustice. To add further confusion, within the world of artificial intelligence and machine learning, similar concepts are commonly described using the word fairness [82]. It may be assumed that, as an inequity is the presence of an inequality due to unfairness or injustice, fairness is therefore the presence of equity. However, without an explicit definition of this, this must be assumed by the reader.

### 4.1. Recommendations

Authors and organisations may have a narrow or broad understanding of which inequalities result from unfairness or injustice, ranging from only those due to very specific differences between groups to defining all health inequalities as inherently unfair and therefore inequities [83]. Furthermore, issues of fairness and unfairness are a product of geography and cultural and political beliefs and are subject to change over time. This may be a reason why many authors did not specifically describe their findings as inequitable. Intersectionality means that splitting inequalities into ‘fair’ inequalities and ‘unfair’ inequities can be a complex decision and heavily dependent on author and societal beliefs, which can also change over time. Different healthcare systems will have different funding models, expectations and tolerance towards inequalities and inequities. Policy makers should promote consistency in approach when evaluating innovations in care provision where equity might be a factor. In practice, many of the studies included in the review are merely quantifying the difference that exist between population groups, and the causes of these differences are left to future research. To assist future researchers or policy makers interpreting their studies, authors should

(1) State their definition of inequality, inequity or disparities (if these terms are used);
(2) State if they consider the differences between their study populations to be fair or unfair (or if they have made no judgements on this);
(3) If they consider the differences to be unfair, state why (and if appropriate, when) they consider this inequality to be unfair (and therefore an inequity).

### 4.2. Limitations

This review considered two components of inequalities and inequity—geographical and socioeconomic—and it should be recognised that there are a multitude of other types of inequalities (e.g., gender, sex, race). While this review has included studies which focused on these other types of inequalities, we have chosen to not analyse any measures of inequality or inequity bar geographic and socioeconomic, and only studies which have included at least of one these have been included. A future review may examine these other inequalities; however, notwithstanding intersectionality, they tend to consist of categorical data such as gender, sex or race, which facilitates straightforward comparisons. In addition, differences in access to acute stroke treatments due to race, gender or sex are generally considered due to unfairness or injustice and therefore should consistently be referenced as an inequity [5]. This review only included studies that were written in the English language, potentially excluding relevant research conducted in other languages. Six studies were rejected at full-text screening based on language, with many others rejected at abstract screening. Finally, this review did not include other acute cardiovascular events that may include commonalities with acute stroke and therefore may share common inequalities and/or inequities.

## 5. Conclusions

Where healthcare systems aim to provide equal access to all individuals irrespective of their personal circumstances, it is vital that the evaluation of programmes like acute stroke care consider impacts on inequality and inequity. Researchers and policy makers should work together to determine relevant measures of inequality/inequity and the most appropriate methods of measuring and categorising them. In addition, researchers should make it clear within their work how they are defining inequality and inequity and what (if any) normative judgements have been made. This will allow readers, policy makers and other researchers to understand the results and conclusions of the research with clarity. In addition, further work is needed to ensure there is a commonality in the use of terms around inequalities and inequities so that future researchers, healthcare funders and policy makers can fully understand and appreciate the full impact of policy decisions. Therefore, when considering acute stroke access to treatments, care must be taken to determine if there are any inequalities or inequities and whether the healthcare system should take these into account when designing pathways.

## Figures and Tables

**Table 1:** Inequalities or inequities included in the studies.

Inequality or Inequity Type	Number of Measures of This Inequality/Inequity
Geographic	81 (58.27%)
Rurality	27 (19.42%)
Geographic regions	24 (17.27%)
Time or distance to hospital	22 (15.83)
Type or designation of hospitals	8 (5.76%)
Socioeconomic	58 (51.73%)
Income	17 (12.23%)
Medical Insurance status	17 (12.23%)
Education	12 (8.63%)
Deprivation	7 (5.04%)
Employment	3 (2.16%)
Other	2 (1.44%)
Total	139 (100%)

## Data Availability

No new data were created or analysed in this study. Data sharing is not applicable to this article.

## Supplementary Materials

The following supporting information can be downloaded at: https://www.mdpi.com/article/10.3390/healthcare13121410/s1, Supplementary Material S1: Search Strategy; Supplementary Material S2: Table of results; Supplementary Material S3: PRISMA-ScR checklist.
