# Supplementary material for: Variations in Methodological Approaches to Measuring Health Inequalities and Inequities: A Scoping Review of Acute Stroke Pathways
Source: Healthcare (Basel). 2025 Jun 12;13(12):1410. doi: 10.3390/healthcare13121410 (PMC12193182; doi:10.3390/healthcare13121410)
Supplement: Supplementary file 1 [file healthcare-13-01410-s001.zip › Supplementary material 1.pdf]

Scopus search criteria

(TITLE-ABS-KEY (stroke*) AND (TITLE-ABS-KEY(thromboly*) OR TITLE-ABS-KEY (thrombec*) OR TITLE-ABS-KEY (reperfu*)) AND (TITLE (socioeconomic*) OR TITLE (social*) OR TITLE (standard* W/3 living) OR TITLE (*income) OR TITLE (*equalit*) OR TITLE (land W/3 tenure) OR TITLE (rac*) OR TITLE (ethnic*) OR TITLE (Nationalit*) OR TITLE (sex*) OR TITLE (gender*) OR TITLE (geograph*) OR TITLE (deprav*))) AND NOT ((INDEXTERMS(animals OR animal)) AND NOT (INDEXTERMS(humans OR human)))

PubMed Search Criteria

((acute stroke[MeSH Terms]) OR (acute strokes[MeSH Terms]) OR stroke*[Title/Abstract]) AND ((mechanical thrombolysis[MeSH Terms]) OR (therapeutic thrombolyses[MeSH Terms]) OR (therapeutic thrombolysis[MeSH Terms]) OR (therapies, thrombolytic[MeSH Terms]) OR (therapy, thrombolytic[MeSH Terms]) OR thromboly*[Title/Abstract] OR thrombec*[Title/Abstract] OR reperfu*[Title/Abstract]) AND ((factor, socioeconomic[MeSH Terms]) OR (factors, socioeconomic[MeSH Terms]) OR (Economic and Social Factors[MeSH Terms]) OR (Socioeconomic Factor[MeSH Terms]) OR (Socioeconomic Characteristics[MeSH Terms]) OR (Characteristic, Socioeconomic[MeSH Terms]) OR (Socioeconomic Characteristic[MeSH Terms]) OR (Social and Economic Factors[MeSH Terms]) OR (Social Inequality[MeSH Terms]) OR (Inequality, Social[MeSH Terms]) OR (Social Inequalities[MeSH Terms]) OR (Standard of Living[MeSH Terms]) OR (Living Standard[MeSH Terms]) OR (Living Standards[MeSH Terms]) OR (High-Income Population[MeSH Terms]) OR (High Income Population[MeSH Terms]) OR (High-Income Populations[MeSH Terms]) OR (Population, High-Income[MeSH Terms]) OR (Land Tenure[MeSH Terms]) OR (Tenure, Land[MeSH Terms]) OR socioeconomic* [TITLE] OR social* [TITLE] OR standard* W/3 living [TITLE] OR *income [TITLE] OR equalit* [TITLE] OR inequalit* [TITLE] OR land W/3 tenure [TITLE] OR geograph* [TITLE] OR deprav* [TITLE] OR region* [TITLE] OR disparit* [TITLE]) AND (NOT (animals [mh]) AND (NOT humans [mh]))
