# Supplementary material for: Variations in Methodological Approaches to Measuring Health Inequalities and Inequities: A Scoping Review of Acute Stroke Pathways
Source: Healthcare (Basel). 2025 Jun 12;13(12):1410. doi: 10.3390/healthcare13121410 (PMC12193182; doi:10.3390/healthcare13121410)
Supplement: Supplementary file 1 [file healthcare-13-01410-s001.zip › Supplementary material 2.pdf]

Citation	Area of interest	Study Design	Source of data	Time period	Geographic Inequalities				Socioeconomic Inequalities					
					Rurality	Distance to hospital	Geographic Regions	Type/Designation of hospital	Income	Medical Insurance Status	Education	Deprivation	Employment	Other
Agarwal et al (2015) (1)	USA	Observational Study*	Nationwide Inpatient Sample database	2003 to 2011	Cat		Cat		Cat	Cat				
Yu et al (2021) (2)	USA	Cross-sectional study*	United States Census Bureau's 2014–2018 American Community Survey, stroke hospitals certified by a state or national body and providing intravenous thrombolysis, and geographic data from a public mapping service	2014 to 2018	Cat	Cat			Cat	Cat				
Mackenhauer et al (2022) (3)	Denmark	Cohort study*	Danish Stroke Registry and the Danish Quality Database for Prehospital Emergency Medical Services	2016 to 2017					Cat		Cat		Cat	
Llanos-Leyton et al (2022) (4)	Colombia	Prospective cohort study*	Collected from a single hospital	2018 to 2019	Cat									

Faysel et al (2019) (5)	USA	Observational Study	New York Statewide Planning and Research Cooperative System (SPARCS)	2010 to 2014				Cat		Cat				
Stecksen et al (2014) (6)	Sweden	Observational Study	Swedish Stroke Register (Riksstroke)	2003 to 2009				Cat			Cat			
Olive-Gadea et al (2023) (7)	Spain	Retrospective longitudinal observational study*	CICAT register	2016		Con								
Barragan-Prieto et al (2022) (8)	Spain	Observational study*	Unified Digital Street Map of Andalucía (CDAU) and picture archiving communication systems (PACS) and medical records	2018 to 2019		Cat								
Yarzebski et al (1996) (9)	USA	Observational study	Hospital Records	1986 to 1991						Cat				
Adeoye et al (2014) (10)	USA	Observational study	US demographic data and 2011 US Medicare Provider and Analysis Review data set	Fiscal year 2011		Cat x2	Cat							
Kapral et al (2020) (11)	Canada	Observational Study	Ontario Road Network File database linked with the 2016 Ontario Acute Stroke	2016		Cat								

			Care Resource Inventory											
Kummer et al (2019) (12)	USA	Observational study	METRONOME (Metropolitan New York Mobile Stroke) registry	Oct 2016 to Sept 2017	Con	Con								
Suolang et al (2021) (13)	USA	Observational study	National Inpatient Sample	2012 to 2018	Cat			Cat	Cat	Cat				
Thompson et al (2022) (14)	New Zealand	Prospective Observational Study*	All 28 New Zealand hospitals and associated rehabilitation and community services caring for patients with acute stroke.	May 1st and October 31st 2018		Con								
Abilleira et al (2017) (15)	Spain	Observational study*	Registry data	2011 to 2015		Cat								
Schuler et al (2023) (16)	Spain	Modelling study	Cartographic information and population statistics were downloaded from publicly available databases from official institutions.	2023			Cat							
Li et al (2019) (17)	China	Survey*	Data from 451 hospitals in different regions of China, by interviewing 484 physicians	Not given			Cat							

			from these hospitals											
Kashani et al (2023) (18)	Canada	Observational study	Canadian OPTIMISE registry	January 2017 and December 2022		Con								
Arrich et al (2008) (19)	Austria	Cohort study*	prospective population-based stroke registry of patients	October 1998 and March 2003							Cat			
Sobral et al (2019) (20)	Portugal	Case-control study*	hospital and primary health care electronic patient records system	2010 to 2015							Cat			Cat x2[a]
Stolz et al (2011) (21)	Germany	Observational study	Hesse Quality Assurance Office	2007 to 2008			Cat							
Freyssenge et al (2018) (22)	France	Modelling study	IGN BDCarto database	Not given		Cat								
Salwi et al (2021) (23)	USA	Prospective observational study*	prospectively-identified, consecutive, acute ischemic stroke patients treated with MT at one academic, comprehensive stroke center	2012 to 2018		Con				Cat		Cat		
Park et al (2021) (24)	South Korea	Observational cross-sectional study*	Acute Stroke Quality Assessment Program (ASQAP)	2013 to 2016				Cat						
Rochemont et al (2022) (25)	France	Prospective cohort study*	three neurology departments of university	June 2011 to October 2014			Cat				Cat	Cat		

			hospitals (Dijon, Burgundy; Fort-de-France, Martinique, Pointe à Pitre, and Guadeloupe) and emergency or medicine departments of three hospitals in French Guiana (Cayenne, Saint-Laurent du Maroni, Kourou).											
Ruiz-Sandoval et al (2018) (26)	Mexico	Observational study	Primer Registro Mexicano de Isquemia Cerebral (PREMIER) study.	January 2005 to June 2006		Cat					Cat			
Ouyang et al (2020) (27)	Worldwide	Observational study	Data prospectively collected from 114 hospitals in nine countries in the HeadPoST study	March 2015 and November 2016			Cat							
Kimball et al (2014) (28)	USA	Observational study	Nationwide Inpatient Sample (NIS) database	2002 to 2008	Cat		Cat		Cat	Cat				
Metcalf et al (2023) (29)	USA	Observational study	Nationwide Inpatient Sample (NIS) database	2019	Cat				Cat	Cat				

Attenello et al (2014) (30)	USA	Observational cohort study	Nationwide Inpatient Sample (NIS) database	2008	Cat				Cat	Cat				
Mehta et al (2021) (31)	USA	Observational cohort study	Nationwide Inpatient Sample (NIS) database	2006 to 2016					Cat	Cat				
Dequatre-Ponchelle et al (2014) (32)	France	Observational study	Hospital Registries	2009 to 2010			Cat							
Choi et al (2015) (33)	USA	Observational study	Office of Statewide Health Planning and Development	2009 to 2010	Cat	Cat								
Grau et al (2021) (34)	Germany	Observational study	DRG statistics and German Index of Multiple Deprivation 2010	2008 to 2017	Cat							Cat		
Dwyer et al (2020) (35)	Australia	Observational study	Admitted Patient Care National Minimum Data Set	1 January 2015 to 8 March 2017	Cat									
Venugopalan et al (2019) (36)	India	Observational study	Indo-US Collaborative Stroke Project	January 2012 to December 2014			Cat							
Dwyer et al (2021) (37)	Australia	Observational study*	Australian Standard Geographical Classification Remoteness Area classification.	2010 to 2015	Cat		Cat					Cat		
Maeda et al (2021)(38)	Japan	Retrospective Analysis*	National DataBase of	2010 to 2015	Con x2			Cat	Con					

			Health Insurance Claims and Specific Health Checkups (NDB).											
Munoz Venturelli et al (2016) (39)	Worldwide	Survey	94 hospitals from 9 middle- and high-income countries completed the questionnaire	26 August 2014 and 1 February 2016	Cat				Cat					
Otsubo et al (2015) (40)	Japan	Observational study	National Claims Database	April 2010 and March 2012			Cat							
Saber et al (2020) (41)	USA	Retrospective study*	Nationwide Inpatient Sample (NIS) database	October 1, 2016, to December 31, 2017			Cat	Cat						
Loccoh et al (2022) (42)	USA	Retrospective cross-sectional study*	Medicare Provider Analysis and Review files	2016 to 2018	Cat		Cat							
Moon et al (2022) (43)	South Korea	Retrospective cohort study	National Health Information Database	January 2010 and December 2019						Cat				
Iosif et al (2012) (44)	Greece	Observational study	four major general public hospitals	1st January 2005 to 31st December 2005		Cat					Cat			
Brinjiki et al (2014) (45)	USA	Observational study	Perspective data base	November 2005 to December 2011	Cat			Cat		Cat				
Buus et al (2022) (46)	Denmark	Observational study*	Danish Stroke Registry	2015 to 2018			Cat		Cat		Cat		Cat	

Ehrlich et al (2021) (47)	USA	Retrospective analysis*	Duke Decision Support Repository	January 2014 to May 2017		Con			Con		Con	Con	Con	
Kleindorfer et al (2009) (48)	USA	Observational Study	MEDPAR database	July 1, 2005 to June 30, 2007	Cat		Cat							
Stein et al (2024) (49)	USA	Retrospective cross-sectional analysis*	National Inpatient Medicare Fee-for-Service datasets	2016 to 2019			Cat							
Gonzalez-Aquines et al (2019) (50)	Mexico	Prospective cohort study*	stroke registry (iReNe)	January to December 2018		Cat					Con			
De Sousa et al (2023) (51)	Europe	Observational Study	Not given	2019 to 2020					Con					
de Havenon et al (2021) (52)	USA	Retrospective, longitudinal analysis*	Nationwide Inpatient Sample (NIS) database	2016 to 2018	Cat		Cat		Cat	Cat				
Chen et al (2019) (53)	China	Observational cross-sectional study*	Nationwide Hospital Discharge Database (NHDD)	1 January 2015 to 31 December 2015			Cat			Cat				
Yuan et al (2023) (54)	China	Cross-sectional study*	Bigdata Observatory Platform for Stroke of China (BOSC)	Jan 1, 2020, to Dec 31, 2020	Cat		Cat x2							
Kim et al (2023) (55)	South Korea	Cross-sectional study*	National Emergency Department Information System (NEDIS) database.	January 2018 to December 2021	Cat							Cat		
Kim et al (2020) (56)	South Korea	Observational Study	Acute Stroke Quality Assessment	March to May 2013 and June		Con								

			Program (ASQAP)	to August 2014										
Crockett et al (2019) (57)	Australia	Prospectively observational study	mechanical thrombectomy registry of Western Australia	August 2016 to July 2018	Cat	Con								
Sobolewski et al (2014) (58)	Poland	Observational Study*	Not given	September 2006 and November 2012	Cat									
Scott et al (1998) (59)	Canada	Modelling study	Statistics Canada	1996		Cat					Cat			
Yan et al (2022) (60)	Canada	Retrospective cohort study*	Interventional neuroradiology registry	2018		Cat								
Eriksson et al (2017) (61)	Sweden	Observational Study	The Swedish Stroke Register (Riksstroke)	2011 to 2012				Cat			Cat			
Perrin et al (2021) (62)	France	Observational Study	STROKE69 study	November 6, 2015, and December 31, 2016		Con						Cat		
Hammond et al (2022) (63)	USA	Retrospective analysis*	National Inpatient Sample	2012 to 2017	Cat		Cat		Cat	Cat				
Hammond et al (2020) (64)	USA	Retrospective cohort study*	National Inpatient Sample	2012 to 2017	Cat		Cat		Cat	Cat				
Sun et al (2024) (65)	USA	Observational Study	National Inpatient Sample	2002 to 2015	Cat		Cat		Cat	Cat				
Walter et al (2021) (66)	Australia	Modelling Study	None	None	Cat									
Total (Cat)					24	13	24	8	14	17	10	6	2	2
Total (Con)					3	9	0	0	3	0	2	1	1	0
Total					27	22	24	8	17	17	12	7	3	2

[a] Other socioeconomic variables are if the patient had a telephone and if the patient received a certain benefit

* Study designs are taken verbatim from their respective study

1. S A, V M, WA J. Outcomes after acute ischemic stroke in the United States: does residential ZIP code matter? England2015 2015-3-15. e001629 p.
2. CY Y, T B, PD P, AP K. Demographic Disparities in Proximity to Certified Stroke Care in the United States. Stroke. 2021;52(8):2571-9.
3. Mackenhauer J, Frischknecht Christensen E, Andersen G, Mainz J, Johnsen SP. Disparities in Reperfusion Therapy and Time Delays among Patients with Ischemic Stroke and a History of Mental Illness. Stroke. 2022;53(11):3375-85.
4. Llanos-Leyton N, Pardo C, Pinilla-Monsalve GD, Arango A, Valderrama J, Pugliese I, et al. Disparities Influencing Functional Outcomes Between Rural and Urban Patients With Acute Stroke. Frontiers in Neurology. 2022;13.
5. MA F, J S, C C, DG S, SR L. Disparities in the Use of Intravenous t-PA among Ischemic Stroke Patients: Population-based Recent Temporal Trends. United States2019 2019-5. 1243-51 p.
6. A S, EL G, K A, B N, M E. Education level and inequalities in stroke reperfusion therapy: observations in the Swedish stroke register. Stroke. 2014;45(9):2762-8.
7. M O-G, N PdlO, T J, S A, X J, P C, et al. Evolution of quality indicators in acute stroke during the RACECAT Trial: Impact in the general population. International journal of stroke : official journal of the International Stroke Society. 2023;18(2):229-36.
8. Barragán-Prieto A, Pérez-Sánchez S, Moniche F, Moyano RV, Delgado F, Martínez-Sánchez P, et al. Express improvement of acute stroke care accessibility in large regions using a centralized telestroke network. European Stroke Journal. 2022;7(3):259-66.
9. J Y, N C, P P, J S, J G, R G. Gender differences and factors associated with the receipt of thrombolytic therapy in patients with acute myocardial infarction: a community-wide perspective. United States1996 1996-1. 43-50 p.
10. Adeoye O, Albright KC, Carr BG, Wolff C, Mullen MT, Abruzzo T, et al. Geographic access to acute stroke care in the United States. Stroke. 2014;45(10):3019-24.
11. MK K, R H, P G, AYX Y, AY J, C M, et al. Geographic Access to Stroke Care Services in Rural Communities in Ontario, Canada. The Canadian journal of neurological sciences Le journal canadien des sciences neurologiques. 2020;47(3):301-8.
12. Kummer BR, Lerario MP, Hunter MD, Wu X, Efraim ES, Omran SS, et al. Geographic analysis of mobile stroke unit treatment in a dense urban area: The new york city metronome registry. Journal of the American Heart Association. 2019;8(24).
13. Suolang D, Chen BJ, Wang N-Y, Gottesman RF, Faigle R. Geographic and regional variability in racial and ethnic disparities in stroke thrombolysis in the United States. Stroke. 2021;52(12):E782-E7.
14. SG T, PA B, JH G, DA C, A D, JN F, et al. Geographic Disparities in Stroke Outcomes and Service Access: A Prospective Observational Study. Neurology. 2022;99(4):e414-26.
15. Abilleira S, Tebé C, de la Ossa NP, Ribó M, Cardona P, Urra X, et al. Geographic dissemination of endovascular stroke thrombectomy in Catalonia within the 2011–2015 period. European Stroke Journal. 2017;2(2):163-70.

16. FAF S, M R, N D-P, J R, T D, MB G, et al. Geographical Requirements for the Applicability of the Results of the RACECAT Study to Other Stroke Networks. Journal of the American Heart Association. 2023;12(20):e029965.
17. J L, J L, Y M, P P, X H, W G. Imbalanced Regional Development of Acute Ischemic Stroke Care in Emergency Departments in China. Emergency medicine international. 2019;2019:3747910.
18. N K, JM O, N S, A Z, A G, JK H, et al. Influence of geography, stroke timing, and weather conditions on transport and workflow times: Results from a longitudinal 5-year Canadian provincial registry. Interventional neuroradiology : journal of peritherapeutic neuroradiology, surgical procedures and related neurosciences. 2023:15910199231196614.
19. Arrich J, Müllner M, Lalouschek W, Greisenegger S, Crevenna R, Herkner H. Influence of socioeconomic status and gender on stroke treatment and diagnostics. Stroke. 2008;39(7):2066-72.
20. S S, I T, R S, AC V, J D, AT G, et al. Late Hospital Arrival for Thrombolysis after Stroke in Southern Portugal: Who Is at Risk? Journal of stroke and cerebrovascular diseases : the official journal of National Stroke Association. 2019;28(4):900-5.
21. E S, GF H, M K, B M. Regional differences in acute stroke admission and thrombolysis rates in the German federal state of Hesse. Germany2011 2011-9. 607-11 p.
22. J F, F R, AM S, L D, N N, K T, et al. Measurement of the potential geographic accessibility from call to definitive care for patient with acute stroke. International journal of health geographics. 2018;17(1):1.
23. Salwi S, Kelly KA, Patel PD, Fusco MR, Mistry EA, Mistry AM, et al. Neighborhood Socioeconomic Status and Mechanical Thrombectomy Outcomes. Journal of Stroke and Cerebrovascular Diseases. 2021;30(2).
24. Park EH, Gil YJ, Kim C, Kim BJ, Hwang S-S. Presence of Thrombectomy-capable Stroke Centers within Hospital Service Areas Explains Regional Variation in the Case Fatality Rate of Acute Ischemic Stroke in Korea. Journal of Preventive Medicine and Public Health. 2021;54(6):385-94.
25. DR R, E M, C M, M P-P, B dT, N S, et al. A Prospective Comparative Study of Health Inequalities and the Epidemiology of Stroke in French Guiana and Dijon, France. Frontiers in public health. 2022;10:849036.
26. Ruiz-Sandoval JL, Briseño-Godínez ME, Chiquete-Anaya E, Arauz-Góngora A, Troyo-Sanromán R, Parada-Garza JD, et al. Public and Private Hospital Care Disparities of Ischemic Stroke in Mexico: Results from the Primer Registro Mexicano de Isquemia Cerebral (PREMIER) Study. Journal of Stroke and Cerebrovascular Diseases. 2018;27(2):445-53.
27. M O, Y Z, X W, L S, L B, T R, et al. Quantifying regional variations in components of acute stroke unit (ASU) care in the international HeadPoST study. Journal of the neurological sciences. 2020;419:117187.
28. MM K, D N, MF W, BL H. Race and income disparity in ischemic stroke care: nationwide inpatient sample database, 2002 to 2008. Journal of stroke and cerebrovascular diseases : the official journal of National Stroke Association. 2014;23(1):17-24.
29. Metcalf D, Zhang D. Racial and ethnic disparities in the usage and outcomes of ischemic stroke treatment in the United States. Journal of Stroke and Cerebrovascular Diseases. 2023;32(12).
30. Attenello FJ, Adamczyk P, Wen G, He S, Zhang K, Russin JJ, et al. Racial and socioeconomic disparities in access to mechanical revascularization procedures for acute ischemic stroke. Journal of Stroke and Cerebrovascular Diseases. 2014;23(2):327-34.

31. Mehta AM, Fifi JT, Shoirah H, Shigematsu T, Oxley TJ, Kellner CP, et al. Racial and socioeconomic disparities in the use and outcomes of endovascular thrombectomy for acute ischemic stroke. American Journal of Neuroradiology. 2021;42(9):1576-83.
32. N D-P, H T, A B, I G-B, R C, P D, et al. Rate of intravenous thrombolysis for acute ischaemic stroke in the North-of-France region and evolution over time. Journal of neurology. 2014;261(7):1320-8.
33. Choi JC, Hsia RY, Kim AS. Regional availability of mechanical embolectomy for acute ischemic stroke in California, 2009 to 2010. Stroke. 2015;46(3):762-8.
34. AJ G, S D, D B, W M, F B, H B. Regional Deprivation, Stroke Incidence, and Stroke Care—An Analysis of Billing and Quality Assurance Data From the German State of Rhineland-Palatinate. Deutsches Arzteblatt international. 2021;118(23):397-402.
35. Dwyer M, Peterson G, Gall S, Kinsman L, Francis K, Ford K, et al. Regional differences in access to acute ischaemic stroke care and patient outcomes. Internal Medicine Journal. 2020;50(8):965-71.
36. VY V, R B, J P, D K, S K, PN S, et al. Regional differences in ischemic stroke in India (north vs. south). International journal of stroke : official journal of the International Stroke Society. 2019;14(7):706-14.
37. Dwyer M, Francis K, Peterson GM, Ford K, Gall S, Phan H, et al. Regional differences in the care and outcomes of acute stroke patients in Australia: An observational study using evidence from the Australian Stroke Clinical Registry (AuSCR). BMJ Open. 2021;11(4).
38. Maeda M, Fukuda H, Matsuo R, Ago T, Kitazono T, Kamouchi M. Regional disparity of reperfusion therapy for acute ischemic stroke in Japan: A retrospective analysis of nationwide claims data from 2010 to 2015. Journal of the American Heart Association. 2021;10(20).
39. Muñoz Venturelli P, Robinson T, Lavados PM, Olavarría VV, Arima H, Billot L, et al. Regional variation in acute stroke care organisation. Journal of the Neurological Sciences. 2016;371:126-30.
40. Otsubo T, Goto E, Morishima T, Ikai H, Yokota C, Minematsu K, et al. Regional variations in in-hospital mortality, care processes, and spending in acute ischemic stroke patients in Japan. Journal of Stroke and Cerebrovascular Diseases. 2015;24(1):239-51.
41. H S, K K, V S, S T, GP C, M N, et al. Reperfusion Therapy Frequency and Outcomes in Mild Ischemic Stroke in the United States. Stroke. 2020;51(11):3241-9.
42. EC L, KE JM, Y W, DS K, RW Y, RK W. Rural-Urban Disparities in Outcomes of Myocardial Infarction, Heart Failure, and Stroke in the United States. United States2022 2022-1-25. 267-79 p.
43. J M, J S, J L, HJ J, H K, J A, et al. Sex and Economic Disparity Related to Reperfusion Therapies for Patients with Acute Ischemic Stroke in South Korea across a 10-Year Period: A Nationwide Population-Based Study Using the National Health Insurance Database. International journal of environmental research and public health. 2022;19(5).
44. C I, M P, E S, A G. Social factors influencing hospital arrival time in acute ischemic stroke patients. Neuroradiology. 2012;54(4):361-7.
45. W B, AA R, HJ C. Socioeconomic disparities in the utilization of mechanical thrombectomy for acute ischemic stroke. United States2014 2014-5. 979-84 p.
46. Buus SMO, Schmitz ML, Cordsen P, Johnsen SP, Andersen G, Simonsen CZ. Socioeconomic Inequalities in Reperfusion Therapy for Acute Ischemic Stroke. Stroke. 2022;53(7):2307-16.

47. Ehrlich ME, Han B, Lutz M, Ghorveh MG, Okeefe YA, Shah S, et al. Socioeconomic Influence on Emergency Medical Services Utilization for Acute Stroke: Think Nationally, Act Locally. Neurohospitalist. 2021;11(4):317-25.
48. Kleindorfer D, Xu Y, Moomaw CJ, Khatri P, Adeoye O, Hornung R. US geographic distribution of rt-PA utilization by hospital for acute ischemic stroke. Stroke. 2009;40(11):3580-4.
49. Stein LK, Maillie L, Erdman J, Loebel E, Mayman N, Sharma A, et al. Variation in US acute ischemic stroke treatment by hospital regions: Limited endovascular access despite evidence. Journal of NeuroInterventional Surgery. 2023.
50. A G-A, AC C-P, M C-N, G P-V, F G-R. Contribution of Onset-to-Alarm Time to Prehospital Delay in Patients with Ischemic Stroke. Journal of stroke and cerebrovascular diseases : the official journal of National Stroke Association. 2019;28(11):104331.
51. Aguiar de Sousa D, Wilkie A, Norrving B, Macey C, Bassetti C, Tiu C, et al. Delivery of acute ischaemic stroke treatments in the European region in 2019 and 2020. European Stroke Journal. 2023;8(3):618-28.
52. De Havenon A, Sheth K, Johnston KC, Delic A, Stulberg E, Majersik J, et al. Acute ischemic stroke interventions in the United States and racial, socioeconomic, and geographic disparities. Neurology. 2021;97(23):E2292-E303.
53. H C, L S, N W, Y H, Y L, M D, et al. Analysis on geographic variations in hospital deaths and endovascular therapy in ischaemic stroke patients: an observational cross-sectional study in China. BMJ open. 2019;9(6):e029079.
54. J Y, ZK L, X X, M L, Y L, LD W, et al. Age and geographic disparities in acute ischaemic stroke prehospital delays in China: a cross-sectional study using national stroke registry data. The Lancet regional health Western Pacific. 2023;33:100693.
55. KH K, YS R, SD S, SJ K. Association between neighborhood socioeconomic status and mechanical thrombectomy for acute ischemic stroke: A nationwide multilevel observational study. Academic emergency medicine : official journal of the Society for Academic Emergency Medicine. 2023;30(9):918-26.
56. Kim JY, Lee K-J, Kang J, Kim BJ, Kim S-E, Oh H, et al. Acute stroke care in Korea in 2013-2014: National averages and disparities. Journal of Korean Medical Science. 2020;35(20).
57. MT C, N J, AJ H, AHY C, TP S, TJ P, et al. Air retrieval for clot retrieval; time-metrics and outcomes of stroke patients from rural and remote regions air-transported for mechanical thrombectomy at a state stroke unit. Journal of clinical neuroscience : official journal of the Neurosurgical Society of Australasia. 2019;70:151-6.
58. P S, W S, A S, M G. Are there differences between rural and urban populations in long-term outcome after systemic cerebral thrombolysis in a hospital located in an agricultural region? Australia2014 2014. 2867 p.
59. Scott PA, Temovsky CJ, Lawrence K, Gudaitis E, Lowell MJ. Analysis of Canadian population with potential geographic access to intravenous thrombolysis for acute ischemic stroke. Stroke. 1998;29(11):2304-10.
60. Y Y, K H, S A, E G, A T, J M, et al. Access to Endovascular Thrombectomy for Stroke in Rural Versus Urban Regions. The Canadian journal of neurological sciences Le journal canadien des sciences neurologiques. 2022;49(1):70-5.
61. M E, EL G, B N, B S, K A. Acute stroke alert activation, emergency service use, and reperfusion therapy in Sweden. Brain and behavior. 2017;7(4):e00654.

62. A P, J F, J H, K T, A T, O G, et al. Are there socio-economic inequities in access to reperfusion therapy: The stroke 69 cohort. Revue neurologique. 2021;177(9):1168-75.
63. Hammond G, Waken RJ, Johnson DY, Towfighi A, Joynt Maddox KE. Racial Inequities Across Rural Strata in Acute Stroke Care and In-Hospital Mortality: National Trends Over 6 Years. Stroke. 2022;53(5):1711-9.
64. Hammond G, Luke AA, Elson L, Towfighi A, Joynt Maddox KE. Urban-Rural Inequities in Acute Stroke Care and In-Hospital Mortality. Stroke. 2020;51(7):2131-8.
65. Sun P, Zheng L, Lin M, Cen S, Hammond G, Joynt Maddox KE, et al. Persistent Inequities in Intravenous Thrombolysis for Acute Ischemic Stroke in the United States: Results from the Nationwide Inpatient Sample. medRxiv. 2023.
66. Walter S, Fassbender K, Easton D, Schwarz M, Gardiner F, Langenberg F, et al. Stroke care equity in rural and remote areas - novel strategies. Vessel Plus. 2021;5.
